# Suppressions of Serotonin-Induced Increased Vascular Permeability and Leukocyte Infiltration by *Bixa orellana* Leaf Extract

**DOI:** 10.1155/2013/463145

**Published:** 2013-10-03

**Authors:** Yoke Keong Yong, NurShahira Sulaiman, Muhammad Nazrul Hakim, Gwendoline Ee Cheng Lian, Zainul Amirudin Zakaria, Fauziah Othman, Zuraini Ahmad

**Affiliations:** ^1^Department of Human Anatomy, Faculty of Medicine and Health Sciences, Universiti Putra Malaysia (UPM), 43400 Serdang, Selangor, Malaysia; ^2^Department of Biomedical Science, Faculty of Medicine and Health Sciences, Universiti Putra Malaysia (UPM), 43400 Serdang, Selangor, Malaysia; ^3^Department of Chemistry, Faculty of Science, Universiti Putra Malaysia, 43400 Serdang, Selangor, Malaysia

## Abstract

The aim of the present study was to evaluate the anti-inflammatory activities of aqueous extract of *Bixa orellana* (AEBO) leaves and its possible mechanisms in animal models. The anti-inflammatory activity of the extract was evaluated using serotonin-induced rat paw edema, increased peritoneal vascular permeability, and leukocyte infiltrations in an air-pouch model. Nitric oxide (NO), indicated by the sum of nitrites and nitrates, and vascular growth endothelial growth factor (VEGF) were measured in paw tissues of rats to determine their involvement in the regulation of increased permeability. Pretreatments with AEBO (50 and 150 mg kg^−1^) prior to serotonin inductions resulted in maximum inhibitions of 56.2% of paw volume, 45.7% of Evans blue dye leakage in the peritoneal vascular permeability model, and 83.9% of leukocyte infiltration in the air-pouch model. 57.2% maximum inhibition of NO and 27% of VEGF formations in rats' paws were observed with AEBO at the dose of 150 mg kg^−1^. Pharmacological screening of the extract showed significant (*P* < 0.05) anti-inflammatory activity, indicated by the suppressions of increased vascular permeability and leukocyte infiltration. The inhibitions of these inflammatory events are probably mediated via inhibition of NO and VEGF formation and release.

## 1. Introduction 

Inflammation is a complicated physiological and pathophysiological process that involves the release of various types of mediators, for example, histamine, serotonin, bradykinin, and prostaglandin [1]. The mediators activate endothelial cells resulting in vasodilation, increase in vascular permeability, edema formation, and leukocyte infiltrations at the site of inflammation [2]. The gap formation between endothelial cells causes fluid and macromolecules to leak into the surrounding tissue leading to edema formation. In addition, neutrophil-endothelium interaction leads to leukocytes migration and contribute to a more persistent increase in permeability. Therefore, the inhibition of the release of mediators or their subsequent effects is a potential strategy to control inflammation. 

*Bixa orellana* L. of Bixaceae family, commonly known as annatto and as Kesumba in Malay, is a plant native to the Central and Southern American rain forest [3]. *B. orellana* is widely used traditionally as a remedy for headaches and blood disorders, as an antiemetic, and to alleviate thirst [4]. Malaysian natives use its leaves for the treatment of gastric ulcers and stomach discomforts, whereas in Peruvian medicine decoction of the leaves is used to treat prostate disorders as well as inflammation [5]. 

Previous phytochemical investigations have revealed that the presence of flavonoid bisulfates, phytol, polyprenol, stigmasterol, and sitosterol in *Bixa orellana* leaves [6, 7] and of an essential oil comprising mainly sesquiterpenes with ishwarane identified as the major compound [8]. These phytochemicals may contribute to the pharmacological activities of the plant. Previous pharmacological studies have revealed that *Bixa orellana*'s leaves and branches extracts were effective at neutralizing the effects of snake venoms [9]. Extracts from different parts of its leaves and seeds have displayed antimicrobial [10–12], antileishmanial, and antifungal activity [13], neuropharmacolocical, anticonvulsant, analgesic, antidiarrhoeal, anti-oxidant and antibacterial activity [14], and antiplasmodial activity [15].

Even though most of *B. orellana*'s bioactivities have been well documented, there is little scientific evidence available on its anti-inflammatory activity. The present study was designed to evaluate the anti-inflammatory effects of *B. orellana* in rats using several animal models. Our aim was mainly to elucidate its inhibitory effects on increased vascular permeability and increased leukocyte infiltration seen during acute inflammation.

## 2. Methodology

### 2.1. Plant Material

 The fresh leaves of *Bixa orellana* were collected from around Universiti Putra Malaysia, and the correct botanical identity was confirmed and identified by the Phytomedicinal Herbarium, Institute of Biosciences, Universiti Putra Malaysia, Selangor, where a voucher specimen has been deposited (no. NL16, *Bixa orellana*). The plant leaves were dried under 60°C in the oven and powdered.

### 2.2. Preparation of Plant Extract

Plant extract was prepared according to previous study [16]. Briefly, the leaves powders were soaked in distilled water based on the ratio one part of powder to twenty part of distilled water (1 g : 20 mL), kept in water bath at 40°C for 24 hours, filtrated, and freeze-dried, giving the aqueous extract (yield 8.5%, w/w). In all experiments, the dosage was recorded as the mass of extract/kg (body weight of rats).

### 2.3. Drugs and Chemicals

5-Hydroxytryptamine (5-HT), evans blue dye, wright's stain, ABTS solution, BSA, protease inhibitor, 10x PBS and mianserin were purchased from Sigma Chemical Co. Ltd., Malaysia. Nitric/nitrate assay kit was purchased from Roche, and Murine VEGF ELISA kit was purchased from Bio Vision, USA.

### 2.4. Experimental Animals

Male *Sprague-Dawley* rats (200–250 g) housed at Animal House in Faculty of Medicine and Health Sciences with access to food and water *ad libitum* were used in these experiments. All experimental designs and procedures were approved by the Animal Care and Use Committee (ACUC), Faculty of Medicine and Health Sciences, Universiti Putra Malaysia.

### 2.5. Anti-Inflammatory Assay

#### 2.5.1. Serotonin-Induced Hind Paw Edema in Rat

Acute inflammation was produced by the subplantar injection of 0.1 mL of 0.2 mg/mL concentration [17] of serotonin in distilled water into the footpad [18]. The AEBO at 50 and 150 mg/kg was given orally for four days and 60 min before serotonin injection on the fourth day. Another group of rats was orally administered with 1 mg/kg of mianserin as a standard reference. Control group received distilled water. The volume of edema was measured by plethysmometer (Model 7140, Ugo Basile, Italy). The measures were determined at 0 min (before serotonin injection) and 60, 120, 180, 240, and 300 min later.

#### 2.5.2. Serotonin-Induced Peritoneal Vascular Permeability on Rats

The method of peritoneal vascular permeability was modified from Whittle [19] following Carvalho et al. [20]. The rats were intravenously injected with 10 mL/kg of 1% Evans blue dye solution in normal saline, followed by intraperitoneal injection of serotonin. AEBO (50 and 150 mg/kg), mianserin (1 mg/kg), and distilled water were given orally for four consecutive days and 1 h prior to the injection of Evans blue on the fourth day. Twenty minutes after injection of serotonin, the rats were sacrificed. Peritoneal exudates were collected after being washed with 5 mL of normal saline, and then they were centrifuged at 2000 rpm for 10 min. The absorbance of the supernatant was read at 610 nm using a spectrophotometer. 

#### 2.5.3. Serotonin-Induced Air-Pouch Model in Rats

The assay was performed according to Martin et al. [21]. AEBO (50 and 150 mg/kg), mianserin (1 mg/kg), and distilled water (control group) were administered orally for 7 d. The air pouch was induced by subcutaneous injection of 20 mL of filtered air on back of rat on the first day after first treatment. On the third and sixth day, an additional 10 mL of filtered air was injected again to maintain the pouch. On day 6, the air pouch of all groups was injected with 1% (10 mg/mL) of serotonin suspended in 1 mL of distilled water and left it for 48 hours. Then, the rats were killed, and the pouch was flushed out with 5 mL of saline. Fluid removed from the pouch was collected then counted using automated hematology analyzer (Sysmex KX-21) for total leukocyte count and also for Wright's staining.

#### 2.5.4. Determination of NO Level from Serotonin-Induced Rat Paws

NO was measured by method of Moshage et al. [22] with using Nitrite/Nitrate Colorimetric Kit (Roche). The sum of nitrite (NO_2_^−^) and nitrate (NO_3_^−^) was evaluated as indicator of NO level. Briefly, five grams of paw tissue was taken and after 60 min injected with serotonin. Tissues were rinsed in 3 mL of ice-cold distilled water and deproteinized with sulfosalicylic acid (35%). Next, it was homogenized it and supernatant was obtained. 100 *μ*L aliquots of sample were mixed with 100 *μ*L of Griess reagent, followed by spectrophotometric measurement at 540 nm. The calibration curve was made with sodium nitrite and potassium nitrate in distilled water (linear range 0–100 *μ*M). 

#### 2.5.5. Serotonin-Induced VEGF Expression in Rat Paws 

 Measurement of VEGF expression was done by using Murine VEGF ELISA kit (BioVision, USA). Briefly, the rats paw inflammation was produced by subplantar injection of 0.2 mg/mL concentration of serotonin after receiving four days of treatment. After 5 hours, rats were killed by cervical dislocation to obtain 0.3 g of paw tissue. The tissue was stored in 4.5 mL of lysis buffer with 1.5 mL of protease inhibitor followed by homogenization and then centrifuged at 10000 rpm for 10 minutes to obtain the supernatant. Sample was analyzed according to protocol of the kit. After 35 minutes incubation time, colour development was monitored with ELISA plate reader (SpectraMAX) at 405 nm with wavelength correction set at 650 nm. 

### 2.6. Statistical Analysis

All values in the figures and test are expressed as mean ± SEM. The *n* represents the number of animals studied. Statistical analysis of data was performed by one-way analysis of variance (ANOVA) and was further analyzed using Dunnet's test. *P* value less than 0.05 (*P* < 0.05) was considered significant.

## 3. Result

### 3.1. Effect of AEBO on Serotonin-Induced Paw Edema

**Figure 1:**
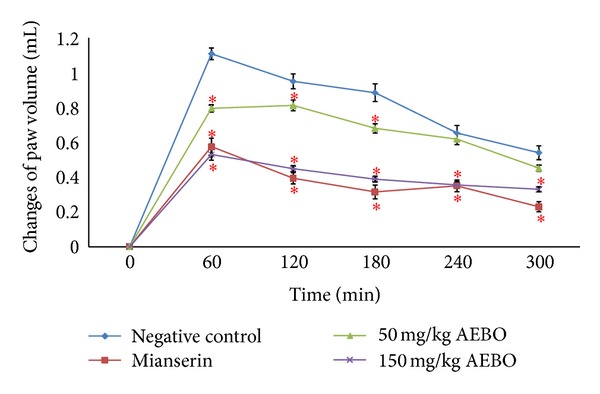
Effect of AEBO (50 and 150 mg/kg) and mianserin (1 mg/kg) on paw volume development elicited by serotonin in the rat. The results are expressed as mean ± SEM (*n* = 6) rats. *P < 0.05, compared with the negative control group at the same time.

Rat's footpad became edematous soon after administrated of serotonin and reached its peak at 60 min (1.11 ± 0.03 mL) (Figure 1). However, AEBO treatment (50 mg/kg and 150 mg/kg) caused significant (*P* < 0.05, resp.) reduction of serotonin-induced paw edema in rats at 1–5 h posttreatment. However, AEBO treatment at 50 mg/kg did not (*P* < 0.05) reduce the paw edema at 4 and 5 h, although it did reduce swelling at 1–3 h.

### 3.2. Effect of AEBO on Serotonin-Induced Peritoneal Vascular Permeability on Rats

**Figure 2:**
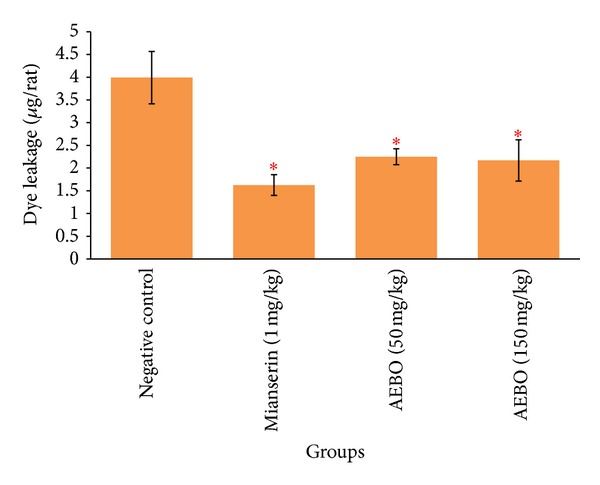
Effect of AEBO on serotonin-induced peritoneal vascular permeability in rats. The results are expressed as mean ± SEM (*n* = 6) rats. *P < 0.05, compared with the negative control group.

As shown in Figure 2, vascular permeability was significantly increased in the peritoneum of the nontreated rats (3.9915 ± 0.5766 *μ*g rat^−1^). However, AEBO at 50 and 150 mg/kg significantly decreased (*P* < 0.05) the vascular permeability which was induced by serotonin compared with the nontreated group (2.25 ± 0.18 *μ*g rat^−1^ and 2.17 ± 0.45 *μ*g rat^−1^, resp.). Mianserin-treated group also showed significant decrease (1.6258 ± 0.2278 *μ*g rat^−1^).

### 3.3. Effect of AEBO on Serotonin-Induced Air-Pouch Model in Rats

**Figure 3:**
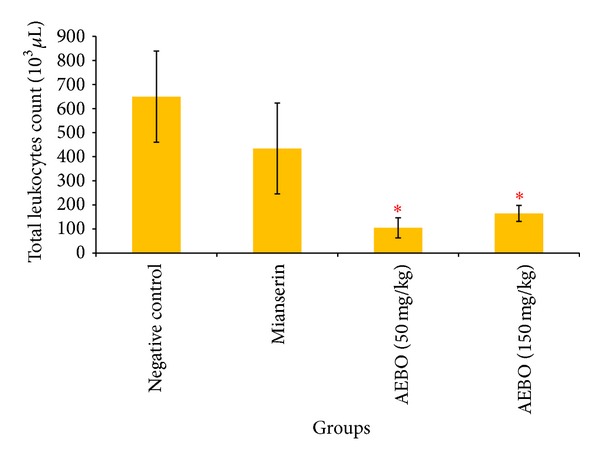
Effect of AEBO (50 and 150 mg/kg) and mianserin (1 mg/kg) on serotonin-induced leukocyte migration of air-pouch model in rats. The results are expressed as mean ± SEM (*n* = 6) rats. *P < 0.05 versus negative control.

**Figure 4:**
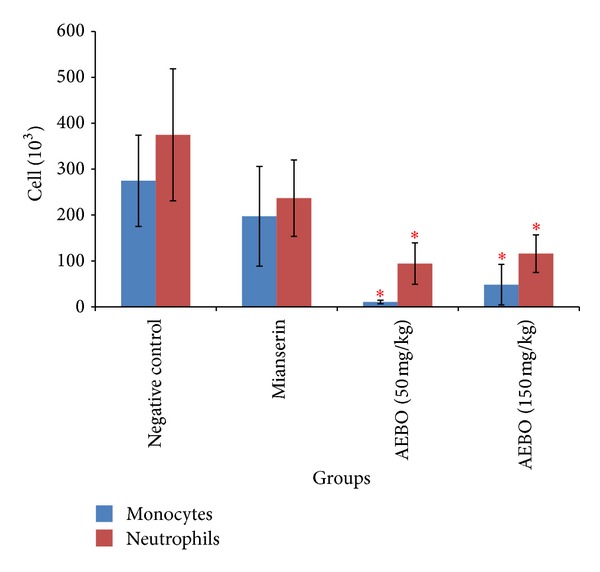
Effect of AEBO (50 and 150 mg/kg) and mianserin (1 mg/kg) on the cell count in exudates from the air-pouch model. The results are expressed as mean ± SEM (*n* = 6) rats. *P < 0.05 versus negative control.

The activity of the AEBO on serotonin-induced air pouch in rats is shown in Figures 3 and 4. AEBO at dose 50 and 150 mg/kg elicited significant (*P* < 0.05) reduction in infiltration of neutrophils (94.25 ± 45.26 × 10^3^ *μ*L and 116 ± 40.98 × 10^3^ *μ*L, resp.) and monocytes (10.5 ± 3.88 × 10^3^ *μ*L and 48.4 ± 44.18 × 10^3^ *μ*L, resp.) into the air pouch compared to control group (374.8 ± 143.73 × 10^3^ *μ*L, neutrophils; 274.6 ± 99.47 × 10^3^ *μ*L, monocytes). There is no significant difference shown between 50 and 150 mg/kg of AEBO. However, there is no significant difference between the mianserin-treated group and the nontreated group.

### 3.4. Effect of AEBO on Serotonin-Induced Rat Paws Nitric Oxide (NO) Level

**Figure 5:**
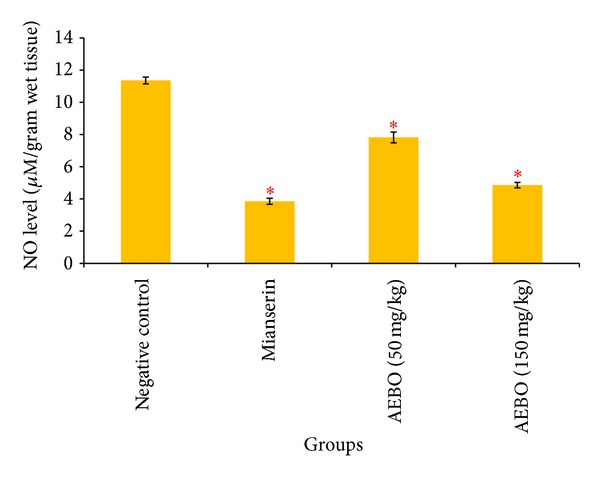
Effect of AEBO on the levels of nitric oxide (NO) production by serotonin-induced NO paw in rats. Each value represents the mean ± SEM of 6 rats. *P < 0.05, compared with the negative control group.

Nitrite and nitrate are the stable end product for nitric oxide. Determination of nitrite and nitrate in fluid represents indirectly measured nitric oxide level. As shown in Figure 5, NO level in paw tissue was significantly increased by serotonin administration (11.35 ± 0.21 *μ*M g^−1^). NO levels were significantly reduced in dose dependent manner in rats treated with 50 and 150 mg/kg of AEBO (7.18 ± 0.33 *μ*M g^−1^, *P* < 0.05; 4.86 ± 0.17 *μ*M g^−1^, *P* < 0.05), respectively. 

### 3.5. Effect of AEBO on Serotonin-Induced VEGF Production in Rat Paws Edema

**Figure 6:**
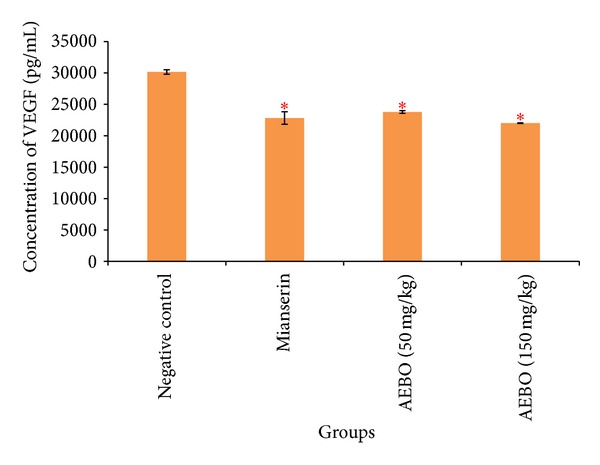
Effect of AEBO (50 and 150 mg/kg) and mianserin (1 mg/kg) on serotonin-induced VEGF expression in rats. The results are expressed as mean ± SEM (*n* = 6). *P < 0.05 versus negative control.

VEGF protein production was measured to show the evidence of increased vascular permeability during acute inflammation. As shown in Figure 6, VEGF expression was significantly increased in hind paw of nontreated rats (30155.28 ± 362.15, resp.). However, 50 and 150 mg/kg of AEBO treated groups showed significant reduction in VEGF expression (*P* < 0.05), from 30155.28 ± 362.15 pg/mL in negative control to 23777.5 ± 207.163 pg/mL and 22011.88 ± 42.81 pg/mL in 50 and 150 mg/kg of AEBO treated groups, respectively. 

## 4. Discussion

In the present study, anti-inflammatory effects of the aqueous extract of *Bixa orellana* leaves were investigated in various related models *in vivo*. The results of the present study indicate that AEBO has indeed played a role as a protective factor against the development of serotonin-induced acute inflammation in various different models of inflammation, that is, serotonin-induced paw edema, serotonin-induced increased peritoneal vascular permeability, and serotonin-induced air-pouch model. Leaves of *Bixa orellana* have been used in the traditional systems of medicine for treating various ailments as antifungal [23], antimicrobial [24], antiplasmodial [15], antimalarial [25], antihypertension, antidiabetes, and antijaundice agent [26]. Recently, our studies also proved that aqueous extract of *B. orellana* leaves was effective against bradykinin and histamine induced acute inflammation [16, 27]. However, the pharmacology of the anti-inflammatory profile of *B. orellana* leaves has still not been fully established. In order to provide more scientific evidence, serotonin was used in current study as another inducer for acute inflammation. 

Acute inflammation can be characterized by vasodilatation, the exudation of protein-rich fluid (plasma), increase of vascular permeability, and cell migration (primarily neutrophil) into the site of inflammation [28]. Among the several methods of acute inflammation, the carrageenan-induced inflammatory response which was described in 1962 [17] in the rat paw has been well established as a valid model to study acute inflammation. To ascertain the effect of the AEBO, serotonin (inflammatory mediator agent) was used as oedemogen [19]. Serotonin is an important inflammation mediator and exerts its effects by initiating vasodilation as well as increasing vascular permeability [29–31]. In the present study, there was a transient increase in paw volume following serotonin injection. The paw volume started to increase beginning at 0 hr and continued up to 1 hr before it gradually decreased. Similar study conducted by Cole et al. [17] also showed a significant increase of paw edema volume at the first hour (*P* < 0.05) after injection of serotonin. AEBO at doses of 50 and 150 mg/kg effectively suppressed the edema and demolished the peak at 1 hr following serotonin injection. Inhibition of 28.27% and 52.18% of acute paw edema was produced at 1 hr by the two doses of AEBO, respectively. Mianserin, a 5-HT receptor antagonist, also showed similar amount of inhibition (48.57%) to 150 mg/kg AEBO.

Evans blue dye or dye-tracer technique [32], in which a dye is injected intravenously, is the most frequently used method for measuring increase in vascular permeability. This vascular permeability assay is a typical model for acute phase of inflammation, where mediators of inflammation released following stimulation lead to dilation of both arterioles and venules and increase vascular permeability [33]. AEBO at oral doses of 50 and 150 mg/kg significantly inhibited increased of peritoneal vascular permeability by 43.43% and 45.68% (*P* < 0.05), respectively.

The serotonin-induced air-pouch model represents both acute and subacute phases of inflammation where in addition to fluid extravasation, infiltration of leukocytes and phagocytic cells can also be studied. Emigration of leukocytes from the microcirculation and their accumulation in inflamed tissue is the hallmark of cellular events during acute inflammation [34]. Serotonin induces a chemotactic activity for the migration of polymorphonuclear granulocytes [35] by raising the intracellular concentration of cyclic GMP [36]. Leukocytes migration is suppressed by specific 5-HT antagonist that blocked the effect of each monoamine [37]. In the present study, total number of leukocytes obtained from pouch's exudate were diminished by the treatment with AEBO (50 and 150 mg/kg) and mianserin. AEBO at both 50 and 150 mg/kg significantly decreased (*P* < 0.05) total leukocyte count (104.8 ± 41.9 × 10^3^ *μ*L; 164.4 ± 33.2 × 10^3^ *μ*L), respectively. However, inhibition of leukocyte infiltration by mianserin was not significantly different from that of control. 

It has been reported that serotonin increases NO level in tissues, and NO contributes to inflammation by increasing vascular permeability that leads to edema formation [38, 39]. 5-HT type II receptor stimulates endothelium-dependent release of NO, as well as NO release by macrophages via iNOS activation [40]. In endothelium, increase in endothelial calcium level leads to NO production through eNOS pathway followed by a cGMP-dependent mechanism to cause increase in vascular permeability [41]. NO level was indeed elevated in the nontreated group in this study, but its level was significantly suppressed (*P* < 0.05) when both AEBO at the doses of 50 and 150 mg/kg were administered. It is likely that AEBO exhibits its acute anti-inflammatory activity through a reduction of NO production. A lower activity of AEBO at dosage 50 mg/kg in the acute anti-inflammatory model and a reduction in NO production may be due to its delayed transport into cells. The result that showed in vascular permeability assay and NO assay looks similar; this can double confirm that AEBO has potent properties in reducing NO production induced by serotonin and may directly reduce the vascular permeability caused by NO.

VEGF was responsible for increased vascular permeability [42]. Vascular permeability can be defined as the movement of solutes, fluids, and molecules between vascular and extravascular compartments [43]. VEGF can induce leak of solutes through several routes via the endothelium including diffusion, interendothelial junctions, fenestrae, caveolae, and the vesicular-vacuolar organelle (VVO). Regarding the study of Kroll and Waltenberger [44], VEGF-A mediates stimulation of endothelial eNOS and inducible iNOS expression which lead to the production of NO via VEGF receptor-2 (VEGFR-2). From the present result obtained, VEGF expression was significantly decreased by both dosages of AEBO (50 and 150 mg/kg) where the VEGF expression was 23777.5 ± 207.163 pg/mL and 22011.88 ± 42.81 pg/mL (*P* < 0.05). Maximum inhibition was observed in 150 mg/kg AEBO-treated group by 27%. These finding suggest that AEBO suppressed increased vascular permeability by reducing VEGF expression as well as NO formation in endothelial cells. The ability of AEBO to reduce VEGF helps to further prevent increased flux of solutes and water normally seen during acute inflammation.

The present study has shown similar trend to that of our previous studies, where AEBO successfully ameliorated bradykinin and histamine induced acute inflammation in animal model [16, 27]. However, the degree of suppression is slightly weaker than histamine. In addition, GC-MS analysis from the previous studies revealed six known phytochemicals, including 2-butanamine, acetic acid, pentanoic acid (valeric acid), phenol, pantolactone, and benzoic acid [16]. Among all the chemical constituents, acetic acid (a short chain fatty acid) was found to be the major compound in the crude extract. Short chain fatty acids (SCFAs) have long been known to regulate immune responses, and it has been reported to possess numerous anti-inflammatory activities, which include suppression of inflammatory cytokine [45, 46], leukocyte adhesion [47], and others [48, 49]. Apart from that, recent study also proved that SCFAs exhibited antimicrobial activity [50]. On the other hand, pentanoic acid, phenol, and benzoic acid have also shown various degrees of biological activities; for instance, those reported include their sedative, anticonvulsant [51], anesthetic/analgesic [52], antiseptic [53], antibacterial [54], and antifungal properties [55]. However, the possibility of the other unknown compounds exhibiting anti-inflammatory properties also cannot be excluded. Hence, it is suggested that major and minor chemical constituents hidden in crude extract may or may not contribute in the suppression of serotonin-induced acute inflammation in the current study. 

## 5. Conclusion

In conclusion, the 50 and 150 mg/kg of AEBO showed anti-inflammatory properties in serotonin-induced acute inflammation. Besides that, it is also shown that AEBO exhibited this effect by reducing vascular permeability induced by serotonin. AEBO also possessed an inhibitory activity on *in vivo* edema production in the rat's paw and reduced the leukocyte migration and also VEGF expression. In addition, anti-inflammatory activity of AEBO may be contributed by the chemical constituents of the aqueous extract. These findings provide additional pharmacological information on the therapeutic efficacy of AEBO. Identification of the potent active compound or component is essential and remained to be clarified in the further studies.
